# Total neoadjuvant therapy or standard chemoradiotherapy for locally advanced rectal cancer: A systematic review and meta-analysis

**DOI:** 10.3389/fsurg.2022.911538

**Published:** 2022-08-26

**Authors:** Zhou Ma, Ling Tan, Zi-lin Liu, Jiang-wei Xiao

**Affiliations:** ^1^ Department of Gastrointestinal Surgery, Clinical Medical College and The First Affiliated Hospital of Chengdu Medical College, Chengdu, China; ^2^ Department of Gastrointestinal Surgery, BaZhong Central Hospital, Bazhong, China; ^3^ Department of Urology, People's Hospital Affiliated to Chongqing Three Gorges Medical College, Chongqing, China

**Keywords:** total neoadjuvant therapy, standard chemoradiotherapy, locally advanced rectal cancer, prognosis, meta-analysis

## Abstract

**Background and Aim:**

The effectiveness of total neoadjuvant therapy (TNT) on patients with locally advanced rectal cancer (LARC) is controversy. This study aims to compare the prognostic value of TNT with standard neoadjuvant chemoradiotherapy (CRT) for LARC.

**Methods:**

We searched databases (Embase [Ovid], Medline [Ovid], PubMed, Cochrane Library, and Web of Science) for articles published between January 1, 2000, and March 10, 2022. Studies on evaluating the effects of TNT and standard CRT on the prognosis of LARC were included. The primary outcomes were overall survival (OS) and disease-free survival (DFS).

**Results:**

19 primary studies, involving 10 randomized controlled trials, 3 prospective studies and 6 retrospective studies, with data on 5,074 patients treated for LARC were included in the meta-analysis. Statistical analyses revealed that, compared with standard CRT, TNT significantly improved OS (hazard ratio [HR]=0.77, 95% confidence interval [CI]=0.65–0.90, *I*^2^ = 30%, *P* = 0.17), DFS (HR = 0.85, 95% CI = 0.74–0.97, *I*² = 11%, *P* = 0.35), distant metastases-free survival (DMFS, HR = 0.76, 95% CI = 0.65–0.90, *I*² = 0%, *P* = 0.50), pathological complete response rate (pCR, OR = 1.89, 95% CI = 1.61–2.22, *I*² = 0%, *P* = 0.47), and R0 resection rate (OR = 1.33, 95% CI = 1.07–1.67, I² = 16%, *P* = 0.28), but local recurrence-free survival (LRFS, HR = 1.12, 95% CI = 0.90–1.39, *I*² = 4%, *P* = 0.37).

**Conclusions:**

Comprehensive literature research shows that TNT showed excellent short-term efficacy in terms of pCR and R0 resection rate while also improved the long-term outcomes of OS, DFS and DMFS, might become a new standard of treatment in patients with LARC. Even so, more studies and longer follow-up were still warranted.

## Introduction

The treatment of locally advanced rectal cancer (LARC, cT3-T4 or N0 or node-positive and M0) is progressing and developing continuously. At present, the accepted standard strategy is 3–4 cycles of neoadjuvant chemoradiotherapy (CRT) before surgery, followed by total mesorectal excision (TME) with or without postoperative chemotherapy. Compared with TME alone or TME with postoperative adjuvant chemotherapy, this standard treatment method shows a better local tumor control rate, R0 resection rate and anal sphincter retention rate (1). Hence, in recent decades, this trimodal therapy has always been the standard of care for LARC (1, 2). However, compared with preoperative neoadjuvant chemoradiotherapy, postoperative adjuvant chemotherapy makes the chemotherapy tolerance and compliance of most patients worse because of factors such as surgical blows and worse nutritional status. Some patients cannot even complete standard postoperative adjuvant chemotherapy, which is a hidden danger for the recurrence and metastasis of LARC (3, 4). In addition, The long-term results of the MRC CR07 and NCIC-CTG C016 trials (5), the EORTC 22921 trial (6), the German CAO/ARO/AIO-94 trial (7) and the Dutch PROCTOR-SCRIPT trial (8) demonstrated that postoperative adjuvant chemotherapy failed to improve overall survival (OS) and disease-free survival (DFS) or had a worse prognosis than without it. Therefore, the goal of many recent studies has been to improve LARC outcomes by modifying treatment strategies.

To achieve a better therapeutic effect, some doctors advocate moving forward with radiotherapy and chemotherapy and even performing surgery after completing all chemotherapy cycles, which referred to as total neoadjuvant therapy (TNT). TNT model includes induction chemotherapy, delivering postoperative adjuvant chemotherapy to pre-CRT, and consolidation, delivering postoperative adjuvant chemotherapy to post-CRT and preoperation. The rationale of TNT was based on the potential to eradicate occult micrometastases before surgery by intensifying neoadjuvant combination chemotherapy (9) and to select the best approach for a given patient by *in vivo* assessment of chemosensitivity (10). Several recent studies have reported that the superiority of TNT is mainly reflected in high rates of tumor pathological complete response (pCR), tumor clinical downstaging, and R0 resection (11–13). In addition, compared with postoperative adjuvant chemotherapy, TNT does not need postoperative chemotherapy, so it can be repaid in advance for patients with protective ileostomy, which shortens the waiting time for ileostomy (14). This results in better patient compliance. A recent meta-analysis reported that TNT remarkably increased the odds of pCR compared with standard CRT (29.9% vs. 14.9%) and DFS (77.6% vs. 67.6%), but there was no statistically significant difference in the proportion of sphincter-preserving surgery or ileostomy (15), However, other meta-analyses reported different pooled results (16, 17). Consequently, the TNT model has received more attention and recognition, but the value of it is still controversial, especially with regard to long-term survival.

Our study retrieved updated and more comprehensive research data and better quality prospective randomized controlled trials, with the purpose of comparing the prognostic effects of TNT and standard CRT for LARC. Once again we scientifically evaluated whether TNT had better clinical significance and value than traditional standard CRT.

## Materials and methods

In the meta-analysis, searching and screening studies, extracting data and quality assessment were following the Meta-Analysis of Observational Studies guidelines (18) and the Preferred Reporting Items for Systematic Reviews and Meta-Analyses statement (19).

### Search strategy and selection criteria

A systematic search was performed based on the following databases: PubMed, Web of Science, Embase (Ovid), Medline (Ovid) and Cochrane Library from January 1, 2000, to March 10, 2022. We used “rectal cancer”, “total neoadjuvant therapy”, “TNT”, “neoadjuvant chemoradiotherapy”, “CRT” and all their relevant keyword variations to search for literatures from above databases (the search strategy is in **Supplementary Table 1**). We restricted our searches to reports published in English. The title and abstract of retrieved articles were screened by two independent reviewers (MZ and TL). All articles about TNT and standard CRT for patients with LARC were accepted for inclusion. All articles of single arm designs, systematic reviews, letters to the editor and publishers, or concerning non-human species, were excluded. Studies that met the inclusion criteria were selected for full-text review. In the event of disagreement, a third reviewer (LZL) was consulted, and the controversial articles were discussed until reached consensus. Eventually, high-quality original studies which compared the prognosis of TNT and standard CRT for LARC were eligible for inclusion.

### Outcomes of interest

The primary outcomes of interest were OS and DFS. The secondary outcomes were LRFS, DMFS, pCR, and R0 resection rate. OS was defined as death from time from surgery to any cause after surgery. DFS was defined as time from surgery to any recurrence after surgery. LRFS was defined as time from surgery to any local recurrence after surgery. DMFS was defined as time from surgery to any distant recurrence. All of local and distant recurrence were confirmed by histological assessment, cytological assessment, or imaging in original studies.

### Data extraction and quality assessment

The following relevant information was extracted from all the included publications: first author, year of publication, country, number of patients, tumor grade, TNT model, years of follow-up and outcome type. If available, the following data were extracted: hazard ratios (HRs), 95% confidence intervals (CIs) and *P* values of OS, DFS, DMFS, and LRFS and odds ratios (ORs), 95% confidence intervals (CIs) and *P* values of pCR and R0 resection. When the literature report OS, DFS, DMFS, and LRFS K-M curves without HRs, Engauge Digitizer (version 10.8) were used to determine the survival rate of the corresponding time points on the curve, followed by the HR calculation table (20). We took the countdown if the HR reported in the literature was TNT vs. standard CRT. All the data were independently extracted by two authors (MZ and TL) and compared for consistency. In the literature quality assessment, RCT literature was assessed based on Cochrane Collaboration's tool (21), and non-RCT literature was assessed based on the Newcastle-Ottawa Scale (NOS) (22). Publication bias was assessed by visual inspection of the symmetry of the funnel plot.

### Statistical analysis

We used the R (version 4.1.0) Meta package for meta-analysis (23). The HRs or ORs with 95% confidence intervals (CIs) of included studies were pooled. Heterogeneity was assessed by using I² index and *P* value. Because of results without heterogeneity, we used fixed effects model for all pooled outcomes. We drew forest plots showing the variation of the study estimates among all studies together with the pooled measure. We assessed publication bias by Egger's regression test for funnel-plot asymmetry. *P* values less than 0.05 were described as significant.

## Results

**Figure 1:**
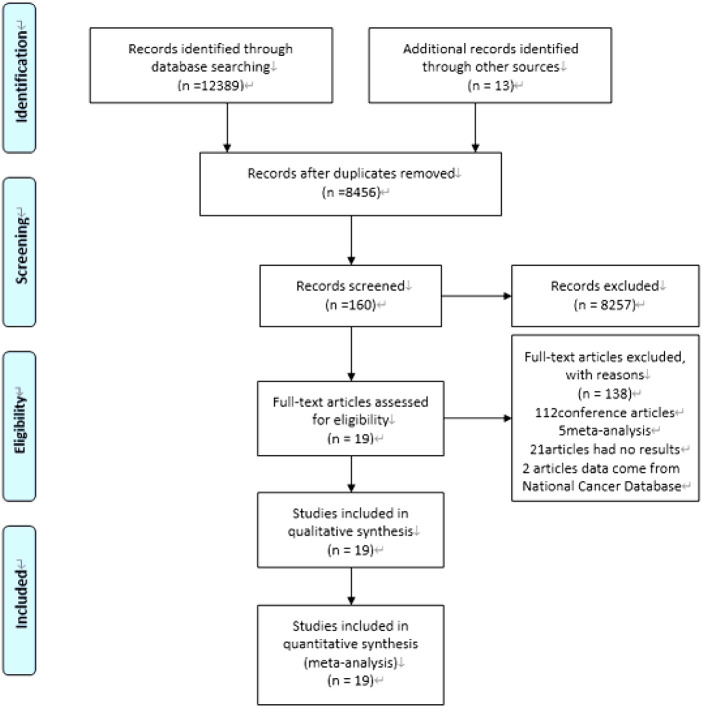
PRISMA flowchart of search strategy and study selection.

Our computer-aided search yielded 8,456 publications from PubMed, Medline (Ovid), Embase (Ovid), Web of Science and Cochrane Library after removing duplicate literature. By screening the titles and reading abstracts, we excluded another 8,298 obviously irrelevant documents. Further full-text screening of 158 publications was carried out, and 138 articles were excluded (Figure 1). Ultimately, this analysis contained 20 articles (14, 24–41), including 10 RCTs (24, 26–29, 31–35) and 3 prospective studies (30, 37, 41) and 6 retrospective studies (14, 25, 36, 38–40). In total, the included studies enrolled 5,074 patients treated for LARC. 2,751 patients received TNT while other 2,323 patients received standard CRT, followed by TME with or without adjuvant chemotherapy. The characteristics of the included studies (number of patients, tumor grade, TNT model, basic characteristics of the study population, etc.) are summarized in Table 1. The risk of bias and literature quality assessment of each included study in the meta-analysis are summarized in **Supplementary Table 3**. For RCTs, the risk of bias tool based on the Cochrane collaboration found that the quality of the included trials met the research standards. For non-RCTs, an NOS score of 7–9 indicates that the quality of the included trials meets the research standards.

**Table 1:** Characteristics of studies included in meta-analysis.

Author, Year	Study design	Country	TNT Mode	Treatment Arms (SCRT vs TNT)	Patients	Clinical Stage	Median follow-up (month)
Cercek, 2018 (14)	Retrospective	America	Induction	Fu/Capecitabine + cRT(50/50.4Gy) + TME + FOLFOX vs. mFOLFOX6 × 8/CAPOXx5/FLOX + Fu/Capecitabine + cRT(50/50.4Gy) + TME	320/328	cT3–T4	40/23
Bahadoer, 2020 (24)	RCT	Netherlands	Consolidation	Capecitabine + cRT(50.4Gy) + TME + CAPOXx8/FOLFOX4 × 12 vs. SRT (25Gy) + CAPOXx6/FOLFOX4 × 9 + TME	450/462	cT4a/b, N2	55.2
Bhatti, 2015 (25)	Retrospective	Pakistan	Induction	Capecitabine + cRT(50.4Gy) +TME *vs.* CAPOX×4 + Capecitabine + cRT(50.4Gy) +TME	61/93	cT3-T4, N+	45
Borg, 2014 (26)	RCT	France	Induction	Bevacizumab + 5-FU + cRT(50.4Gy) + TME + adjuvant chemotherapy vs. Bevacizumab + FOLFOX4 + bevacizumab+5-FU + cRT(50.4Gy) + TME	45/46	T3, N+	-
Chakrabarti, 2021 (27]	RCT	India	Consolidation	Capecitabine + cRT(50/54Gy) +TME + XELOX × 6 vs. SRT (25Gy) + XELOX×2 + TME + XELOX×6	71/69	cT3–T4, N+	NR
Conroy, 2020 (28)	RCT	France	Induction	Capecitabine + cRT(50.4Gy) +TME + mFOLFOX6 × 12/Capecitabinex8 vs. FOLFIRINOXx6 + Capecitabine + cRT(50.4Gy) +TME + mFOLFOX6 × 6/Capecitabinex4	230/231	cT2–T4, N+	46.5
Fernandez-Martos, 2015 (29)	RCT	Spain	Induction	CAPOX + cRT(50.4Gy) +TME + CAPOX *vs.* CAPOX×4 + CAPOX + cRT(50.4Gy) +TME + CAPOX	52/56	cT3-T4, cT3N+, threatened CRM	69
Garcia-A, 2015 (30)	Prospective	America	Consolidation	Fu + cRT(45Gy) + TME vs. Fu + cRT(45Gy) + mFOLFOX6 × 2/4/6 + TME	60/199	cT2–T4, N+	NR
Jin, 2022 (31)	RCT	China	Consolidation	Capecitabine + cRT(50.4Gy) +TME + CAPOX × 6 vs. SRT (25Gy) + CAPOX×4 + TME + CAPOX×2	230/235	cT3–T4, N+	35.0
Bujko, 2016 (32)	RCT	Poland	Consolidation	5-Fu + LV + Oxaliplatin + cRT(50.4Gy) +TME vs. SRT (25Gy) +FOLFOX4 × 3 + TME	254/261	cT3-T4	35
Kim, 2018 (33)	RCT	Korea	Consolidation	Capecitabine + cRT(50.4Gy) + TME + CAPOX/Capecitabine vs. Capecitabine + cRT(50.4Gy) + CAPOXx2 + TME	55/53	cT3–T4, N+	26
Marechal, 2012 (34)	RCT	Belgium	Induction	5-Fu + cRT(45Gy) + TME vs. FOLFOXx2 + 5-FU + cRT(45Gy) + TME	29/28	cT2–T4, N+	NR
Moore, 2017 (35)	RCT	Australia	Consolidation	5-Fu + cRT(45Gy) + TME vs. +5-FU + cRT(45Gy) + Bolus 5-FU + TME	24/25	cT3–T4, N+	NR
Liang, 2019 (36)	Retrospective	China	Consolidation	Cap/CAPOX/FOLFOX + cRT(50.4Gy) + TME + adjuvant Cap/CAPOX/FOLFOX vs. CAP/CAPOX/FOLFOX + cRT + Cap/CAPOX/FOLFOX + TME	80/76	cT3-T4, N0-N2	31
Marco, 2018 (37)	Prospective	America	Consolidation	5-FU + cRT (50.4 Gy) +TME+ FOLFOX vs. 5-FU + mFOLFOX6 × 2/4/6 + cRT (50.4 Gy)	40/171	cT2–T4, N+	59
Markovina, 2017 (38)	Prospective	America	Consolidation	5-FU/Capecitabine + cRT(45Gy) +TME + FOLFOX/CAPOX vs. SRT (25Gy) + mFOLFOX6 + TME	69/69	cT3-T4	54/49
Mojca, 2021 (39)	Retrospective	Slovenia	Induction + Consolidation	Capecitabine-based + cRT(45/50.4/54Gy) + TME + Capecitabine/CAPOX vs. CAPOX/FOLFOX + Capecitabine-based + cRT(45/50.4/54Gy) +CAPOX/FOLFOX + TME	72/89	cT2–T4, N+	NR
Voogt, 2021 (40)	Retrospective	Netherlands	Induction	Capecitabine + cRT(50.4Gy) + TME vs. CAPOX/CAPOX-bevacizumab/FOLFOX + Capecitabine/Tegafur/gimeracil/oteracil + cRT(50.4Gy) +TME	53/53	cT2–T4, N+	NR
Calvo, 2014 (41)	Prospective	Spain	Induction	5FU + RT (50.4Gy) +TME+5FU+ leucovorin vs. FOLFOX4 × 2 + 5FU + cRT(50.4Gy) +TME	128/207	cT3–T4, N+	72.6

RT, radiotherapy; cRT, concurrent radiotherapy; SRT, short-course radiotherapy; TME, total mesorectal excision; CAPOX/XELOX, capecitabine and oxaliplatin; FOLFOX, oxaliplatin, leucovorin and fluorouracil; Fu, fluorouracil; 5-Fu, 5- fluorouracil; RCT, randomized controlled trial; NR, not reporting.

### Primary outcomes

#### OS for TNT vs. standard CRT

Nine (24, 25, 28, 29, 31, 32, 36, 38, 41) of the 19 included studies reported OS data based on TNT and standard CRT; the HRs and 95% CIs of these studies are summarized in Figure 3A. The overall HR was 0.77 (95% CI: 0.65–0.90). The heterogeneity test showed that these trials were not heterogeneous (*I*^2^ = 30%, *P* = 0.17).

**Figure 2:**
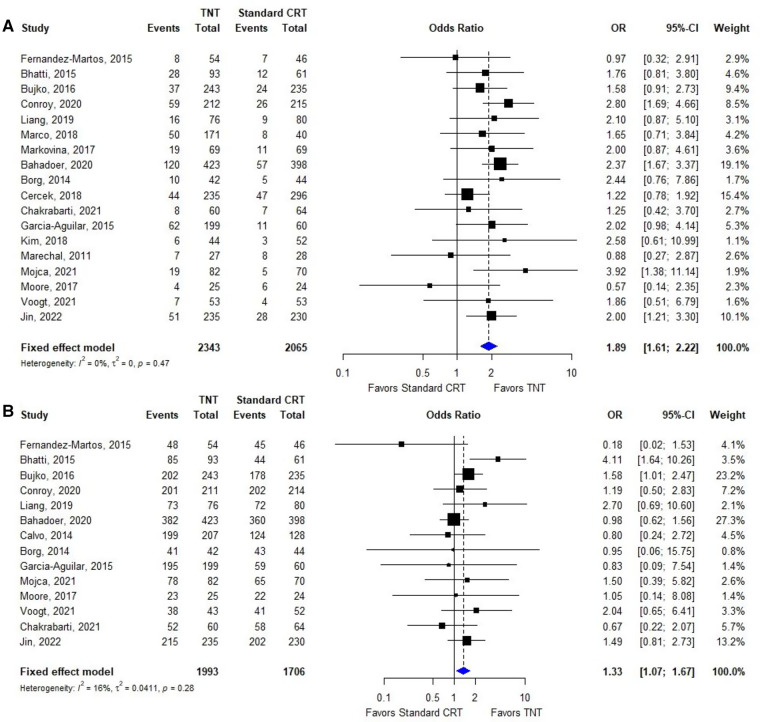
Forest plots for the meta-analyses. (**A**): pCR; (**B**): R0 resection.

#### DFS for TNT vs. standard CRT

**Figure 3:**
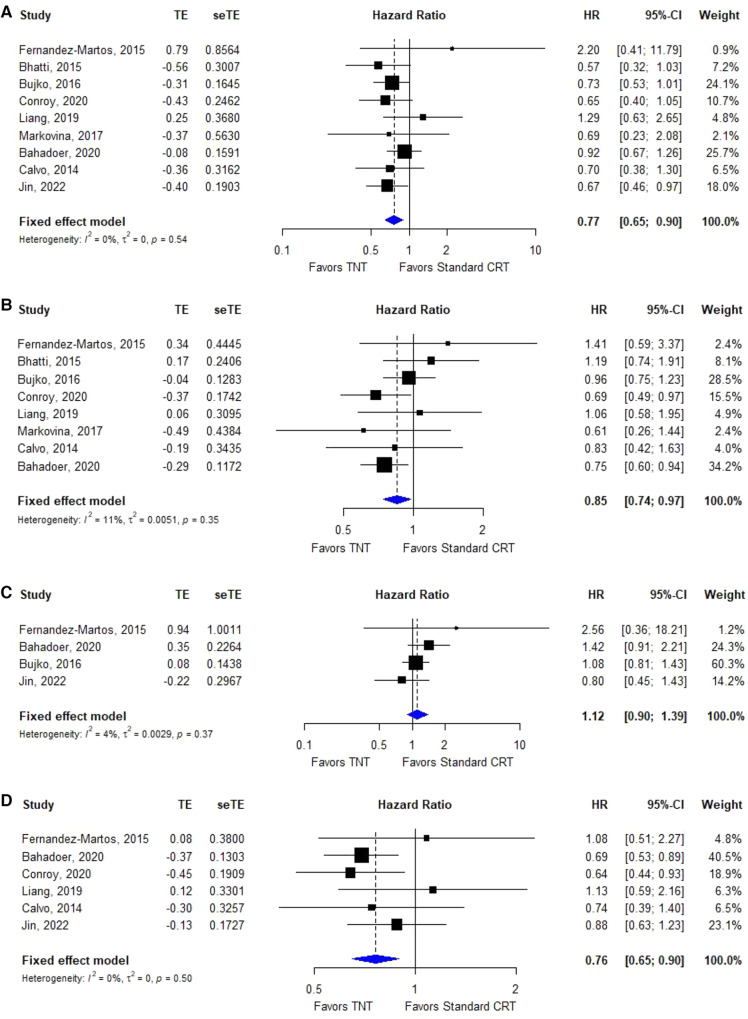
Forest plots for the meta-analyses. (**A**): OS; (**B**): DFS; (C): LRFS; (D): DMFS.

Eight (24, 25, 28, 29, 32, 36, 38, 41) of the 19 included studies reported DFS data based on TNT and standard CRT; the HRs and 95% CIs of these studies are summarized in Figure 3B. The overall HR was 0.85 (95% CI: 0.74–0.97). The heterogeneity test showed that these trials were not heterogeneous (*I*^2^ = 11%, *P* = 0.35).

### Secondary outcomes

#### pCR for TNT vs. standard CRT

Nineteen (14, 24–40) of the 19 included studies reported pCR data based on TNT and standard CRT; the ORs and 95% CIs of these studies are summarized in Figure 2A. The overall OR was 1.89 (95% CI: 1.61–2.22). The heterogeneity test showed that these trials were not heterogeneous (*I*^2^ = 0%, *P* = 0.47).

#### R0 resection for TNT vs. standard CRT

Fourteen (24–32, 35, 36, 39–41) of the 19 included studies reporting R0 data based on TNT and standard CRT; the ORs and 95% CIs of these studies are summarized in Figure 2B. The overall OR was 1.33 (95% CI: 1.07–1.67). The heterogeneity test showed that these trials were not heterogeneous (*I*^2^ = 16%, *P* = 0.28).

#### LRFS for TNT vs. standard CRT

Three (24, 29, 31, 32) of the 21 included studies reported LRFS data based on TNT and standard CRT; the HRs and 95% CIs of these studies are summarized in Figure 3C. The overall HR was 1.12 (95% CI: 0.90–1.39). The heterogeneity test showed that these trials were not heterogeneous (*I*^2^ = 4%, *P* = 0.37).

#### DMFS for TNT vs. standard CRT

Five (24, 28, 29, 31, 36, 41) of the 20 included studies reported DMFS data based on TNT and standard CRT; the HRs and 95% CIs of these studies are summarized in Figure 3D. The overall HR was 0.76 (95% CI: 0.65–0.90). The heterogeneity test showed that these trials were not heterogeneous (*I*^2^ = 0%, *P* = 0.50).

### Publication bias

**Figure 4:**
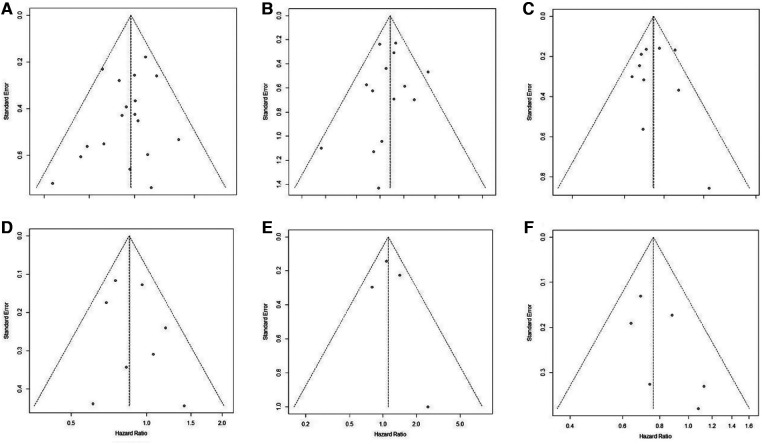
Funnel plot of publication bias in the meta-analysis. (**A**): pCR; (**B**): R0 resection; (**C**): OS; (**D**): DFS; (**E**): LRFS; (**F**): DMFS.

Publication bias was assessed by visual examination of the symmetry of the funnel plot. Our funnel plot showed no publication bias (Figure 4).

## Discussion

This meta-analysis and systematic review retrieved the latest and most comprehensive literature data to compare the efficacy of TNT with standard CRT. In this summary analysis, our research table shows that TNT improves OS, DFS, pCR, R0 resection rate and DMFS in advanced rectal cancer. Unfortunately, TNT does not improve LRFS in advanced rectal cancer.

The standard treatment plan for stage II and III rectal cancer includes neoadjuvant radiotherapy and chemotherapy, followed by radical surgical resection. In addition, some milestone studies have demonstrated the benefits of preoperative CRT. According to reports, the failure rate of local area treatment is <10%. It can significantly improve the radical resection rate of local tumors and the chance of preserving anal sphincter function (5, 42). Therefore, neoadjuvant CRT followed by radical surgical resection has become the standard treatment for rectal cancer. The long-term follow-up results after this treatment showed that the 5-year survival rate for a specific stage was 63% to 77.4% (43, 44). Radical surgery combined with perioperative adjuvant radiotherapy and chemotherapy has reduced the local recurrence rate from 25% to 40% to less than 10%, but the remote recurrence rate was still as high as 25% to 38% (4, 45). The traditional "sandwich" treatment model reduces the local recurrence rate but at the same time highlights the risk of distant metastasis, which affects the long-term survival of patients. In recent years, many clinical studies have completed adjuvant radiotherapy and chemotherapy before surgery, which is called TNT. An increasing number of studies have shown that the TNT model has more advantages than the traditional "sandwich” treatment model. Cercek et al. (14). reported a study of 628 patients with LARC. Among them, 320 patients were treated with traditional concurrent CRT + TME + postoperative adjuvant chemotherapy (CRT + TME + ACT), and 308 patients received TNT. The chemotherapy regimen was FOLFOX or CAPOX. The results showed that the completion rate of radiotherapy and chemotherapy in the TNT group was significantly higher than that of the traditional treatment group (*P* < 0.001), and the distant recurrence rate was also significantly lower in the TNT group. The incidence of complete remission (complete response), including pCR and clinical complete remission (clinical complete response, cCR), was higher than that in the traditional treatment group. Previous studies have shown that increasing pCR may help reduce the risk of recurrence and death. Although not sufficient to replace OS, pCR is considered an important prognostic parameter for the long-term outcome of LARC (46). Recent studies have shown that TNT increases the pCR of LARC, especially for high-risk patients, such as T4 and peripheral resection margin involvement. Fokas et al. (47). showed that, compared with adverse pathological reactions, the cumulative incidence of distant metastases in the pCR of patients was significantly reduced (10.5% vs. 39.6%) and DFS was higher (89.5% vs. 63%). Perhaps pCR can truly be an important indicator of long-term survival for advanced rectal cancer.

Previous studies have shown that TNT can improve the pCR of LARC, further improve the patient's OS and DFS, and reduce distant metastasis (24, 28, 38). Similar results were obtained in our meta-analysis. However, there was no significant difference between TNT and standard CRT in terms of local recurrence-free survival (LRFS). We speculate that it may be due to extension of the preoperative treatment period, some nonresponsive patients have local tumor progression, and the risk of residual tumor is increased. On the other hand, although the proportion of patients with pCR was increased by TNT, the tissue cell microenvironment after tumor regression might differ widely from normal tissues, which was difficult to identify and resect intraoperatively and induced identical local relapse risks between TNT and standard CRT.

Inspiringly, our meta-analysis indicated that TNT had favorable outcomes regarding DFS and DMFS. The primary inadequacy of standard CRT was the difficulty of decreasing the distant relapse rate (48), suggesting that the metastatic potential was not acquired by nongenomic factors but specialized tumor cells that present cancer stem cell properties (49). Cancer stem cells existing in the hematologic and lymphatic systems might lead to high distant relapse for LARC. The reason that TNT significantly increased DMFS might be explained by the ability to eradicate cancer stem cells in the hematologic and lymphatic systems. TNT can intervene in micrometastasis at an earlier stage, reduce tumor cell activity, reduce free tumor cells to a certain extent, and reduce the risk of metastasis and implantation caused by radiotherapy and surgery. Perhaps because of this increased DMFS, DFS was significantly increased by TNT. Meanwhile, OS in the TNT group showed an obvious advantage compared with that in the standard CRT group in our meta-analysis. Reported evidence has indicated that TNT possesses many advantages, such as better compliance with treatment, pCR rate, R0 resection rate, DMFS and DFS. Hence, TNT improved OS logically. However, at the same time, because the chemotherapy time of TNT is longer and the cumulative effect of radiotherapy and chemotherapy toxicity is more obvious, the grade 3–4 toxicity of TNT is usually higher than that of standard CRT (24). The increased grade 3–4 toxicity seems to have no effect on the excellent compliance rate and long-term survival results of the TNT group, but it also needs to attract more attention.

In the TNT model, is the chemotherapy regimen before radiotherapy? Or after? It is controversial and may have a different effect on the patient's prognosis. There are two regimens of induction and consolidation chemotherapy for the TNT regimen of LARC. To date, the two regimens are still controversial. The Spanish GCR-3 trial took 4 cycles of COPAX before CRT as an induction regimen because CAPOX-based CRT achieved pCR rates of 10% to 19% in some studies (29, 50, 51). The results of the Spanish GCR-3 trial showed that TNT had a similar effect on pCR, 5-DFS and 5-OS compared with standard CRT (29). Nevertheless, as an induction chemotherapy regimen in the UNICANCER-PRODIGE 23 trial, 6 cycles of FOLFIRINOX before CRT took full advantage of significantly improved pCR, 3-DFS and 3-year metastasis-free survival rates (28). In the RAPIDO trial (24), the consolidation chemotherapy regimen after short-course radiotherapy consisted of 6 cycles of CAPOX or 9 cycles of FOLFOX4. Except for pCR, the cumulative incidence of disease-related treatment failure and distant metastases were lower with TNT. In a randomized clinical trial in Spain, neoadjuvant short-course radiotherapy followed by 2 cycles of XELOX achieved indistinctive pCR compared with standard CRT (27). Our study conducted a subgroup analysis of induction and consolidation chemotherapy for TNT. The results showed that the induction and consolidation regimens of TNT compared with those of standardized radiotherapy can significantly improve pCR, and the consolidation regimen of TNT, compared with standardized CRT, can improve OS. However, there was no difference in OS between the induction regimen for TNT and standardized CRT (**Supplementary Figure 1**). Therefore, it is necessary to further study the prognostic difference between the induction regimen TNT and the consolidation regimen TNT of LARC. Recently, adding targeted drugs and immunotherapy to induction chemotherapy has been widely studied. Studies have shown that the pCR rate of TNT combined with pembrolizumab is higher than that of TNT alone (31.9% vs. 29.4%) (52), and TNT and cetuximab significantly improved OS (53). Immunotherapy might activate T cells to reach tumors by limiting the interaction of programmed cell death 1 (PD-1) with its ligand PD-L1 and has shown clinical efficacy in patients with tumors. The addition of targeted therapies might further increase pCR rates or reduce metastatic progression for LARC (54). Whether adding immunotherapeutic drugs or targeted drugs will improve and optimize the therapeutic effect during TNT is still inconclusive. More convincing evidence is needed to clarify the clinical application value of the above drugs. Meanwhile, it is necessary to study the molecular mechanism of TNT to achieve personalized treatment for LARC.

TNT has the following advantages over traditional CRT: (1) it can intervene in micrometastasis at an earlier stage; (2) it can reduce tumor burden and staging, as well as increase the R0 resection rate; (3) Avoid it radiotherapy and surgery destroy the original structure of the tumor structure, use the original blood supply of the tumor, increase the drug perfusion rate, and increase the effect of chemotherapy; (4) compared with postoperative radiotherapy and chemotherapy, TNT can control symptoms early and has higher patient compliance and tolerance, ensuring the implementation of a complete course of chemotherapy; (5) it inhibits the stimulation of tumor proliferation caused by surgery; (6) Avoid chemotherapy delays (such as anastomotic leakage) caused by poor recovery of some postoperative patients, and significantly shorten prevention The fistula period of patients with ileostomy improves the quality of life of patients; (7) it reduces tumor cell activity, reduces free tumor cells (to a certain extent), and reduces the risk of metastasis and implantation caused by radiotherapy and surgery; and (8) it does not significantly increase the cost, while the shortening of the chemotherapy cycle can also reduce costs (to a certain extent). However, in theory, TNT has some of the following shortcomings: (1) the preoperative treatment period is prolonged, which increases the short-term potential risk of progression in nonresponsive patients; (2) it affects the body's immune status and reduces the patient's surgical tolerance; and (3) it increases the risk of adverse events during the perioperative period. Of course, there are still many unresolved problems in the TNT model. For example, is it better to use induction chemotherapy or consolidation chemotherapy in the TNT model? What are the reasons for their differences? After the whole course of chemotherapy, the patient's physical condition will be weaker, and the ability to withstand surgical shocks will be worse. How do you balance the choices? After TNT, some patients can achieve pCR. Does this type of patient need surgery? If surgery is not possible, how can this type of patient be effectively screened out? During TNT, do immunotherapy drugs or targeted drugs be added, and what role do they play? These are issues worthy of further in-depth study and discussion.

This meta-analysis and systematic review showed that TNT can improve the OS, DFS, PCR, R0 resection and DMFS of LARC. At the same time, this study has some limitations. First, although 10 randomized trials were included, only 5 randomized trials had long-term follow-up results. Additionally, other phase 1 and phase 2 randomized trials are ongoing. Second, the current research lacks a direct comparison between induction and consolidation programs, and more relevant data should be collected in current ongoing trials in the future.

## Conclusion

Our study has demonstrated that, compared with standard CRT, TNT therapy has excellent short-term efficacy in terms of pCR and R0 resection rate, while also improving OS, DFS, and DMFS for long-term results. However, The safety, effectiveness and clinical economics of the TNT model still require a large number of clinical high-quality research evaluations. Similar to the traditional treatment model, the TNT model is a combination of challenges and opportunities.

## Data Availability

The original contributions presented in the study are included in the article/**Supplementary Material**, further inquiries can be directed to the corresponding author/s.
