# Supplementary material for: Total neoadjuvant therapy or standard chemoradiotherapy for locally advanced rectal cancer: A systematic review and meta-analysis
Source: Front Surg. 2022 Aug 26;9:911538. doi: 10.3389/fsurg.2022.911538 (PMC9458916; doi:10.3389/fsurg.2022.911538)
Supplement: Supplementary file 1 [file Presentation_1_v1.pptx]

## Slide 1
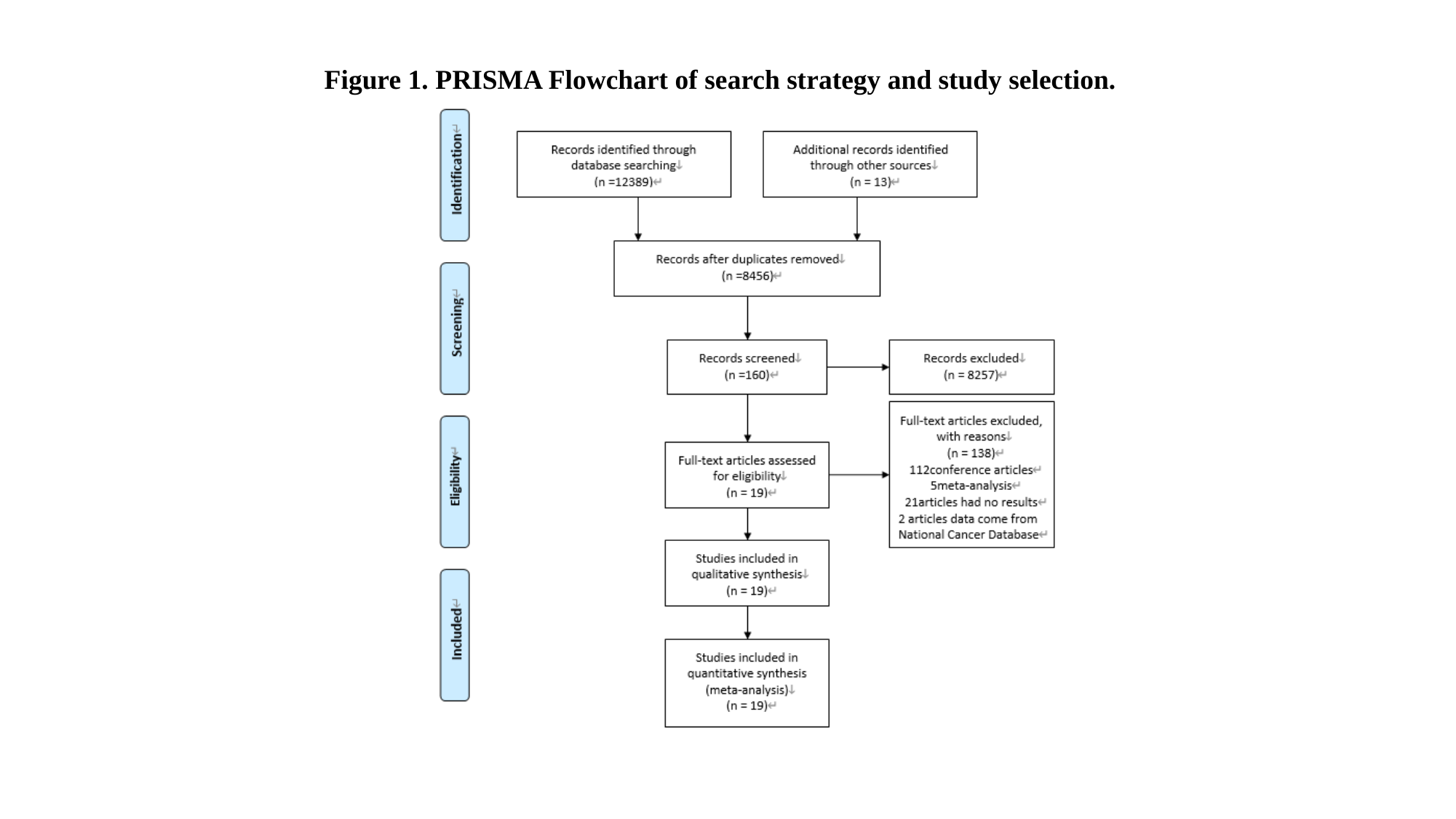

Figure 1. PRISMA Flowchart of search strategy and study selection.

## Slide 2
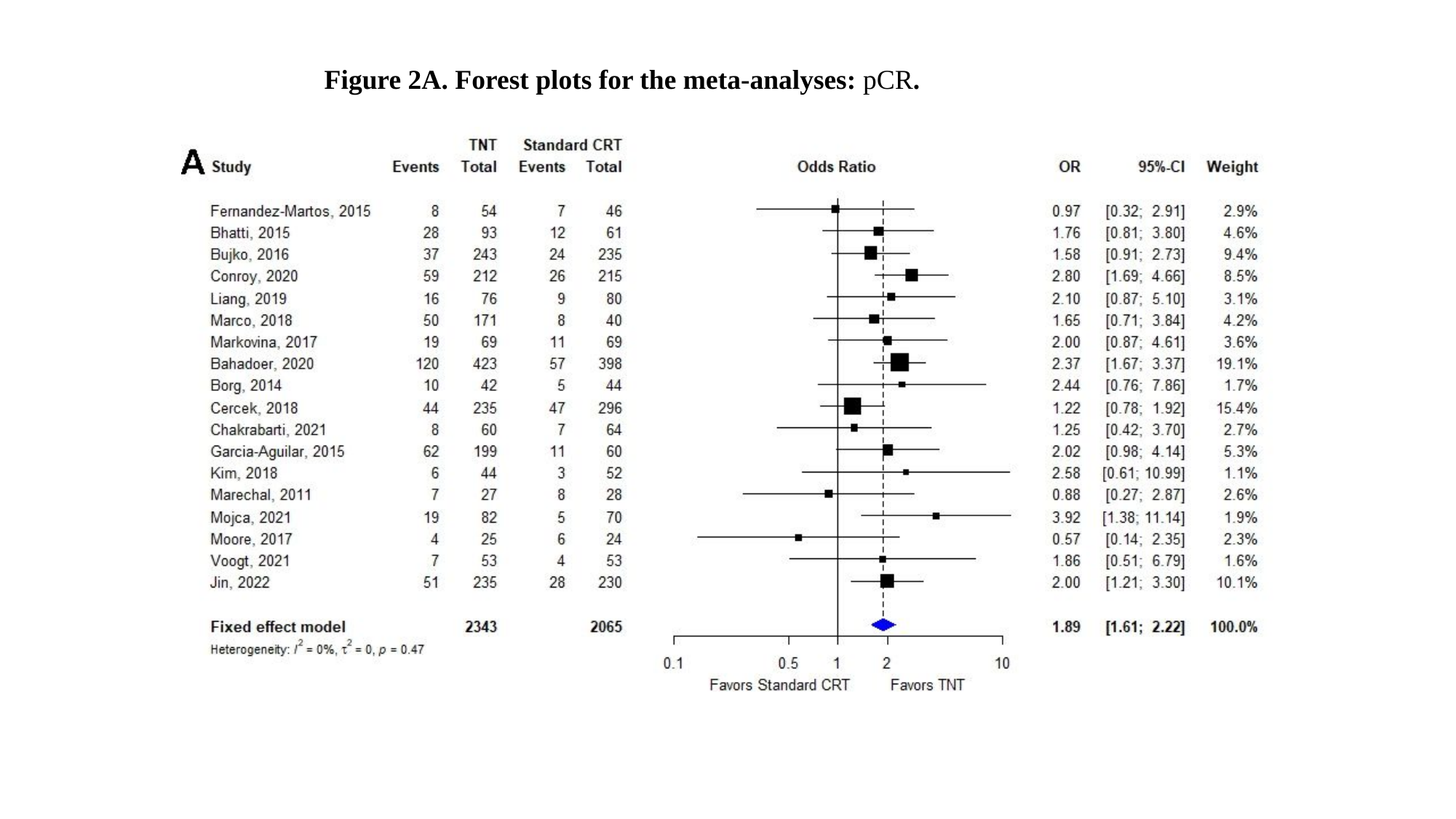

Figure 2A. Forest plots for the meta-analyses: pCR.

## Slide 3
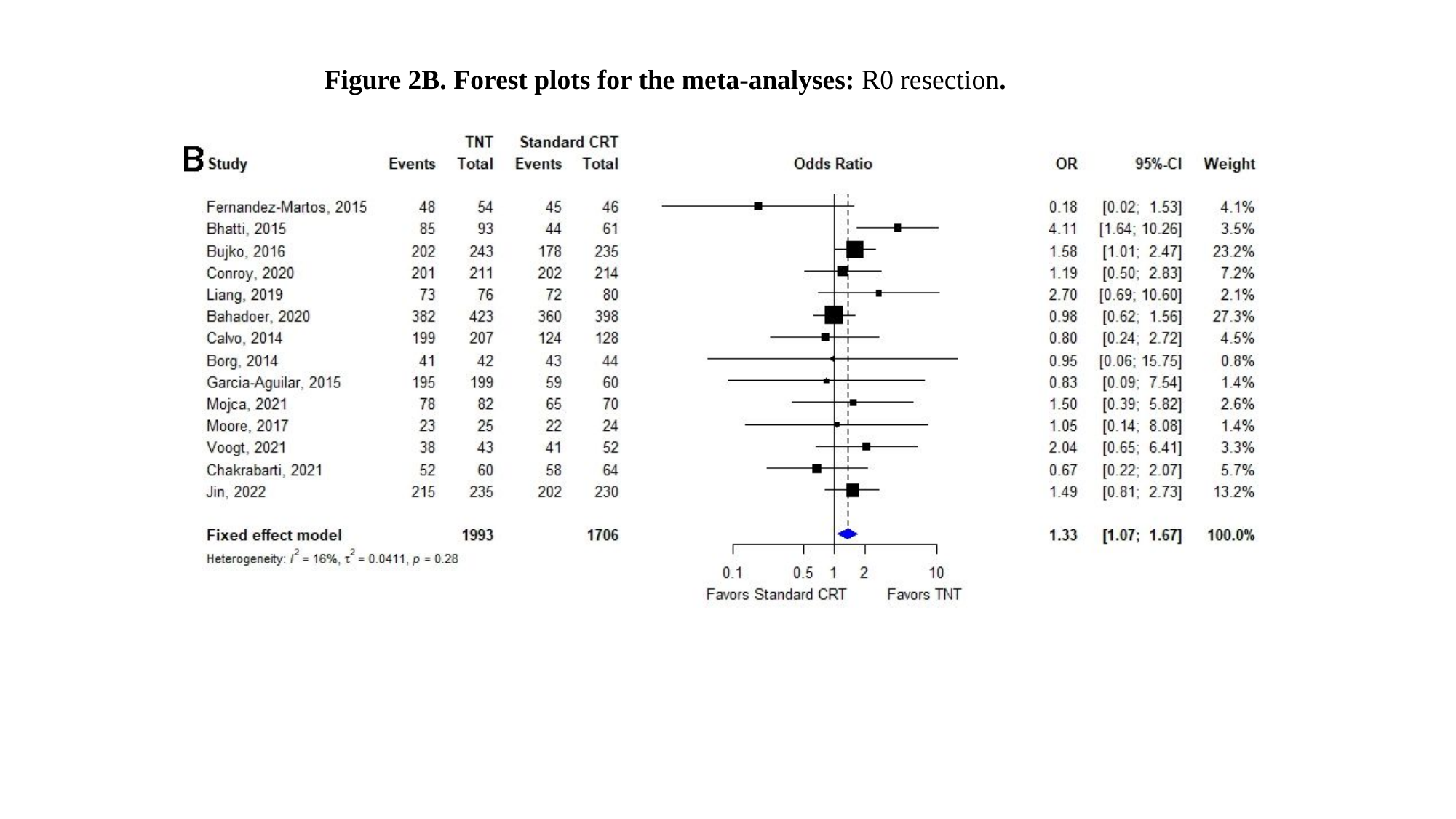

Figure 2B. Forest plots for the meta-analyses: R0 resection.

## Slide 4
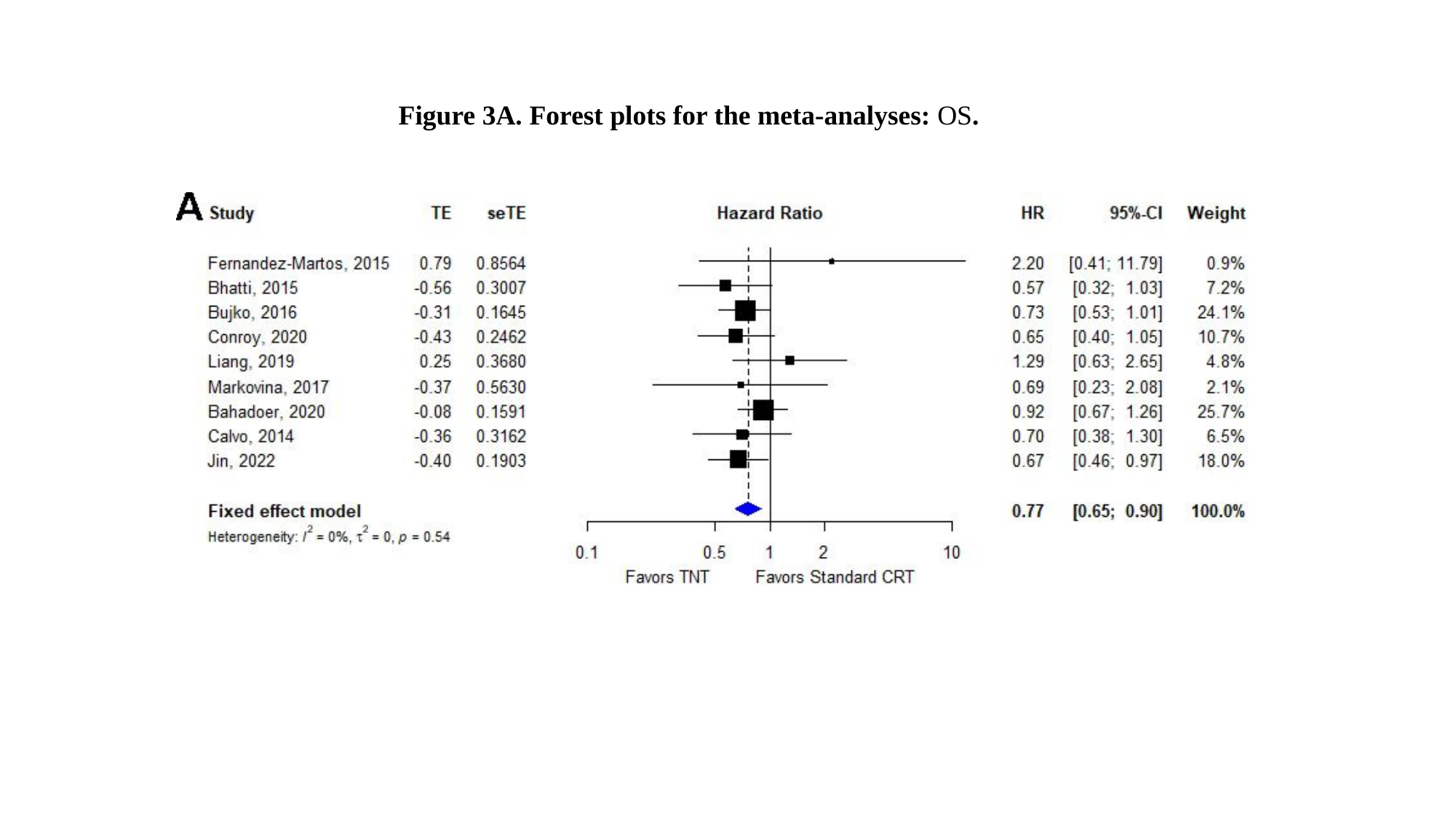

Figure 3A. Forest plots for the meta-analyses: OS.

## Slide 5
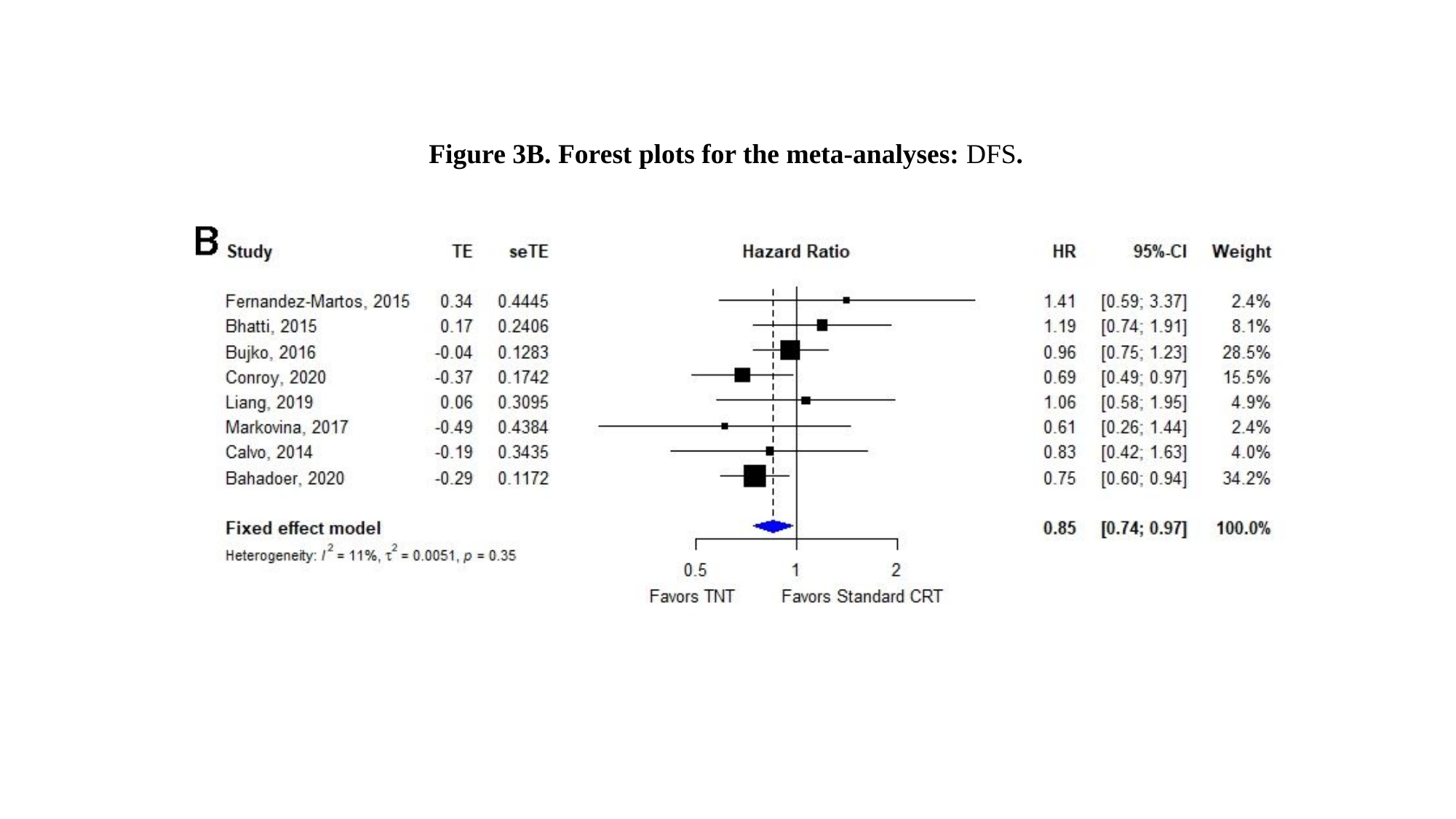

Figure 3B. Forest plots for the meta-analyses: DFS.

## Slide 6
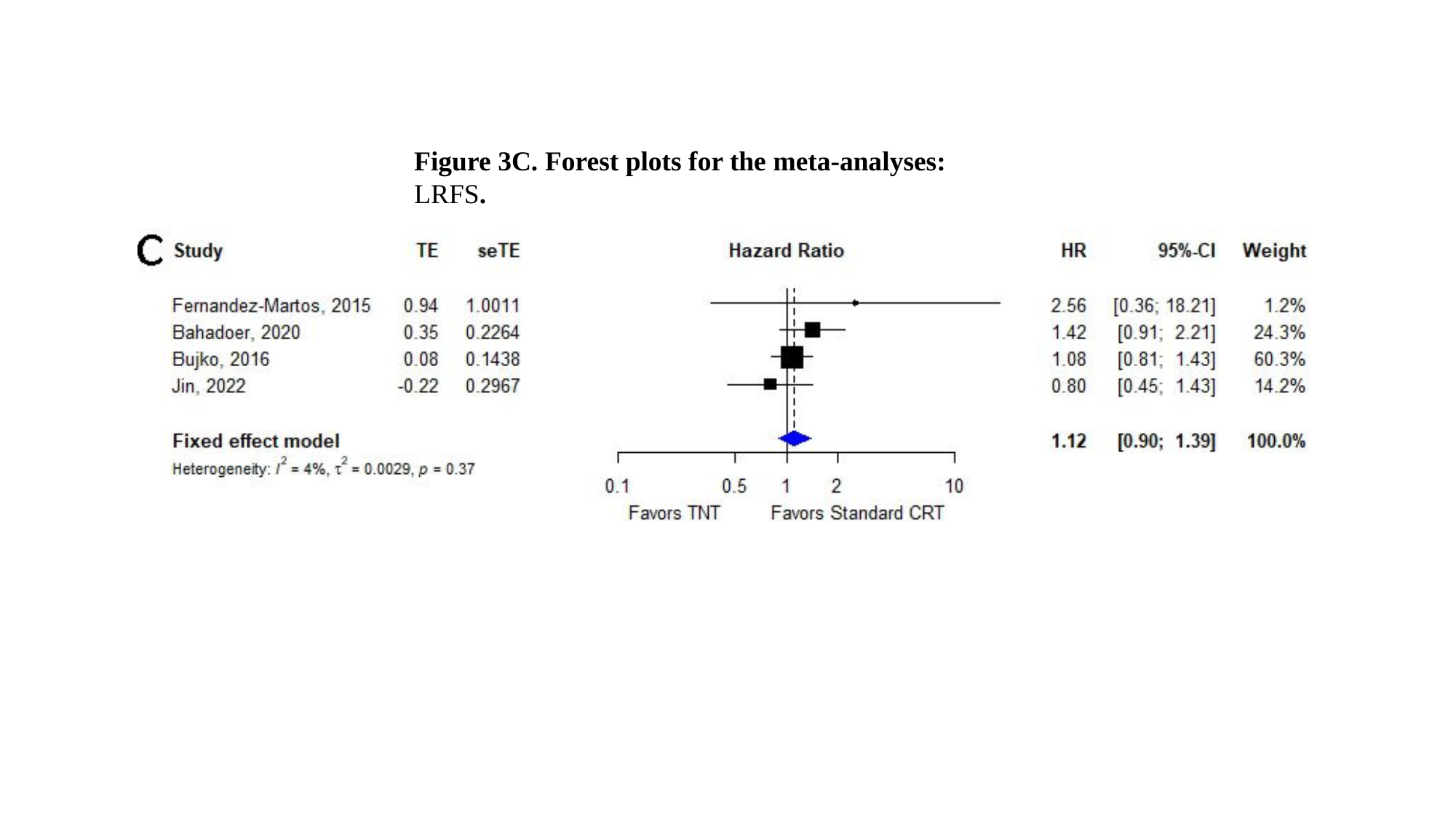

Figure 3C. Forest plots for the meta-analyses: LRFS.

## Slide 7
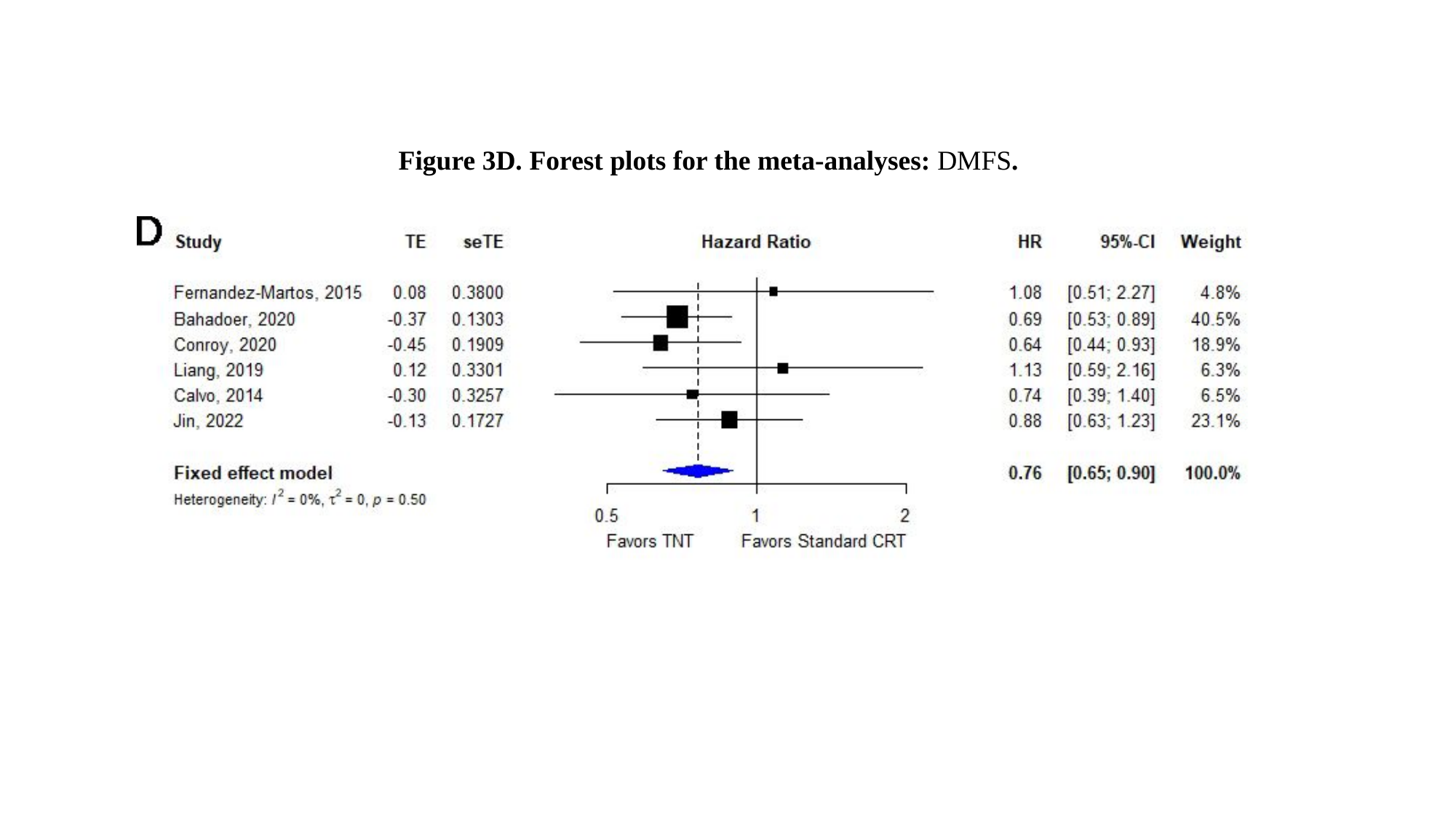

Figure 3D. Forest plots for the meta-analyses: DMFS.

## Slide 8
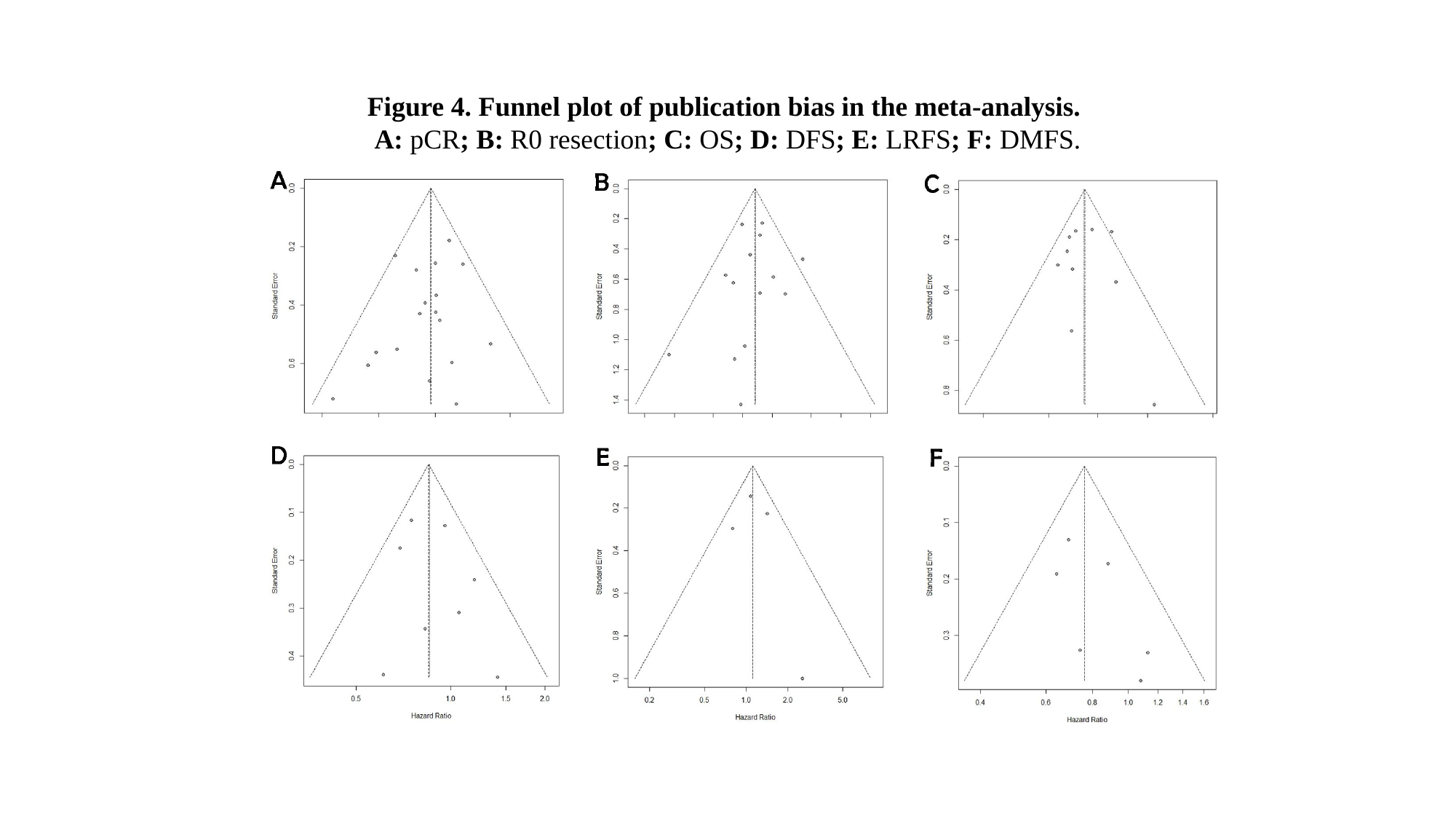

Figure 4. Funnel plot of publication bias in the meta-analysis.
A: pCR; B: R0 resection; C: OS; D: DFS; E: LRFS; F: DMFS.

## Slide 9
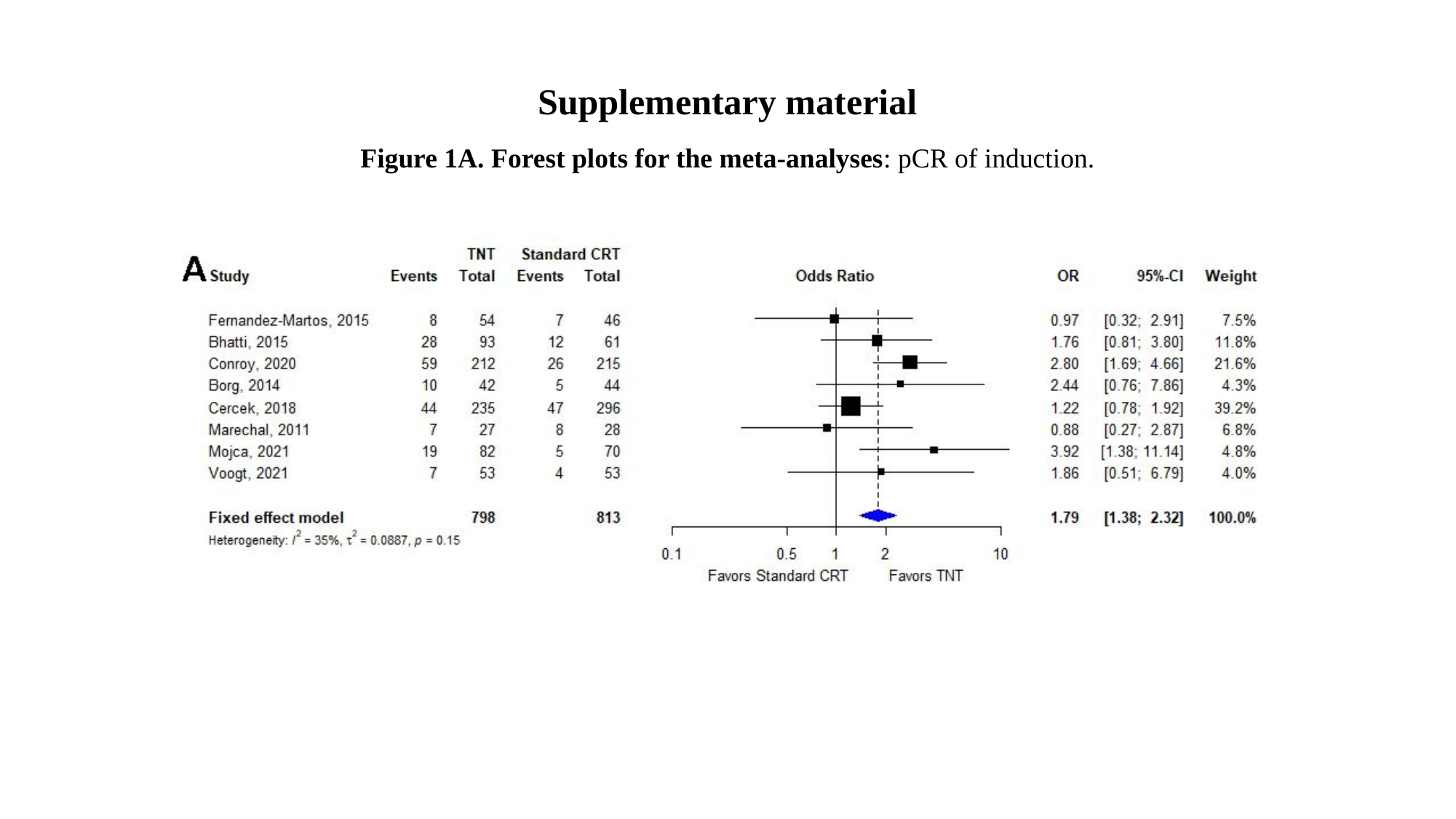

Supplementary material
Figure 1A. Forest plots for the meta-analyses: pCR of induction.

## Slide 10
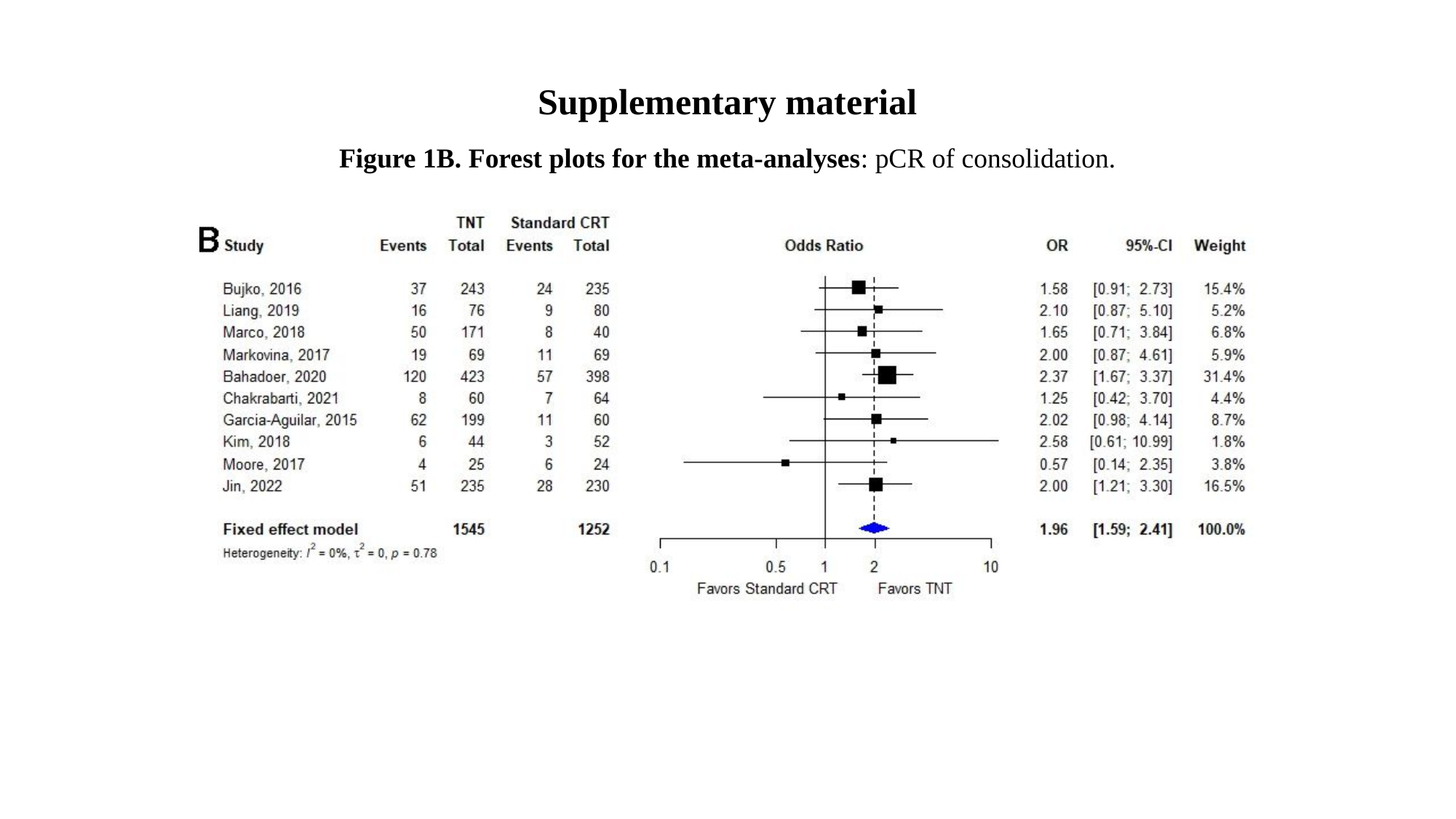

Supplementary material
Figure 1B. Forest plots for the meta-analyses: pCR of consolidation.

## Slide 11
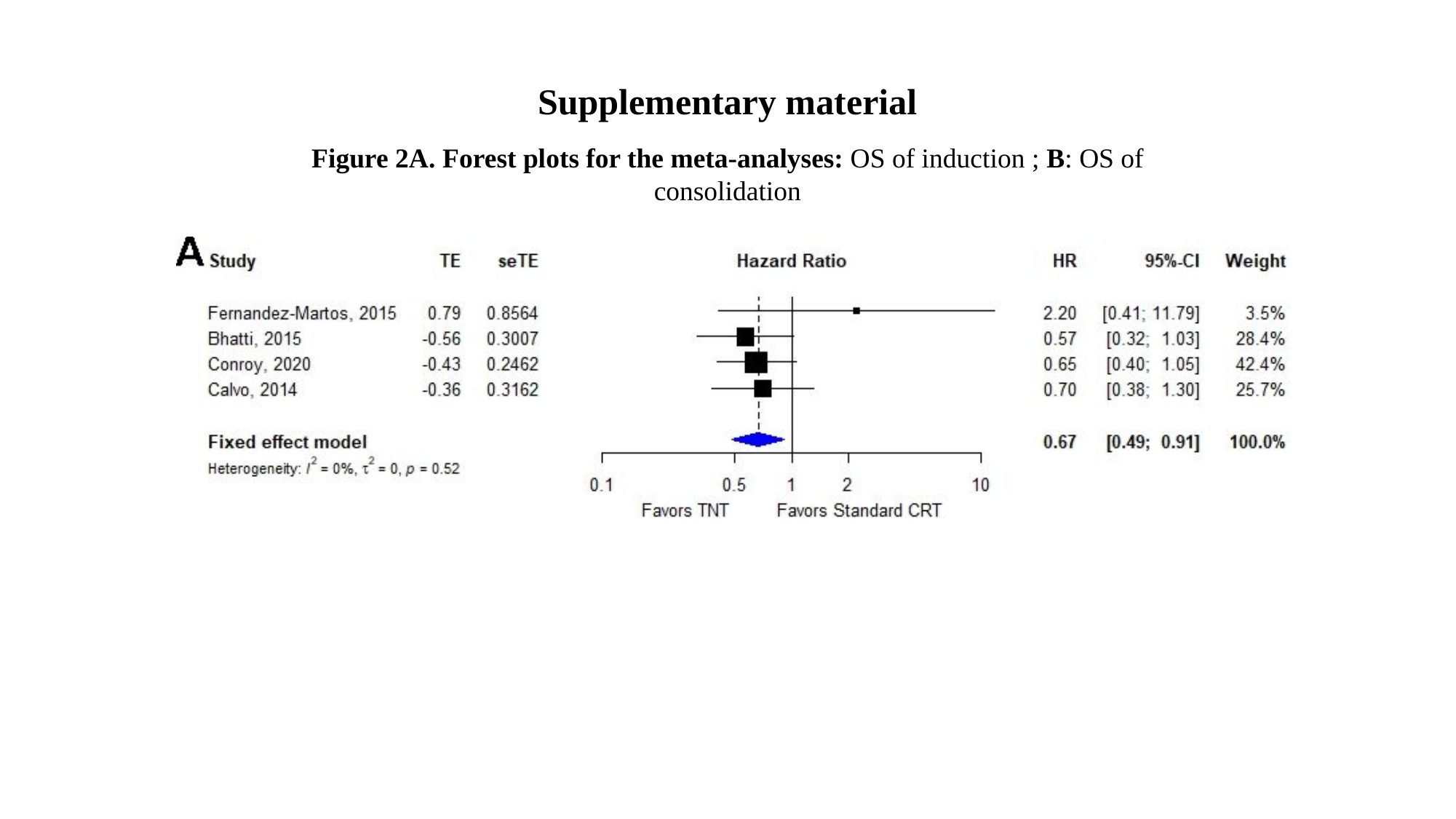

Supplementary material
Figure 2A. Forest plots for the meta-analyses: OS of induction ; B: OS of consolidation

## Slide 12
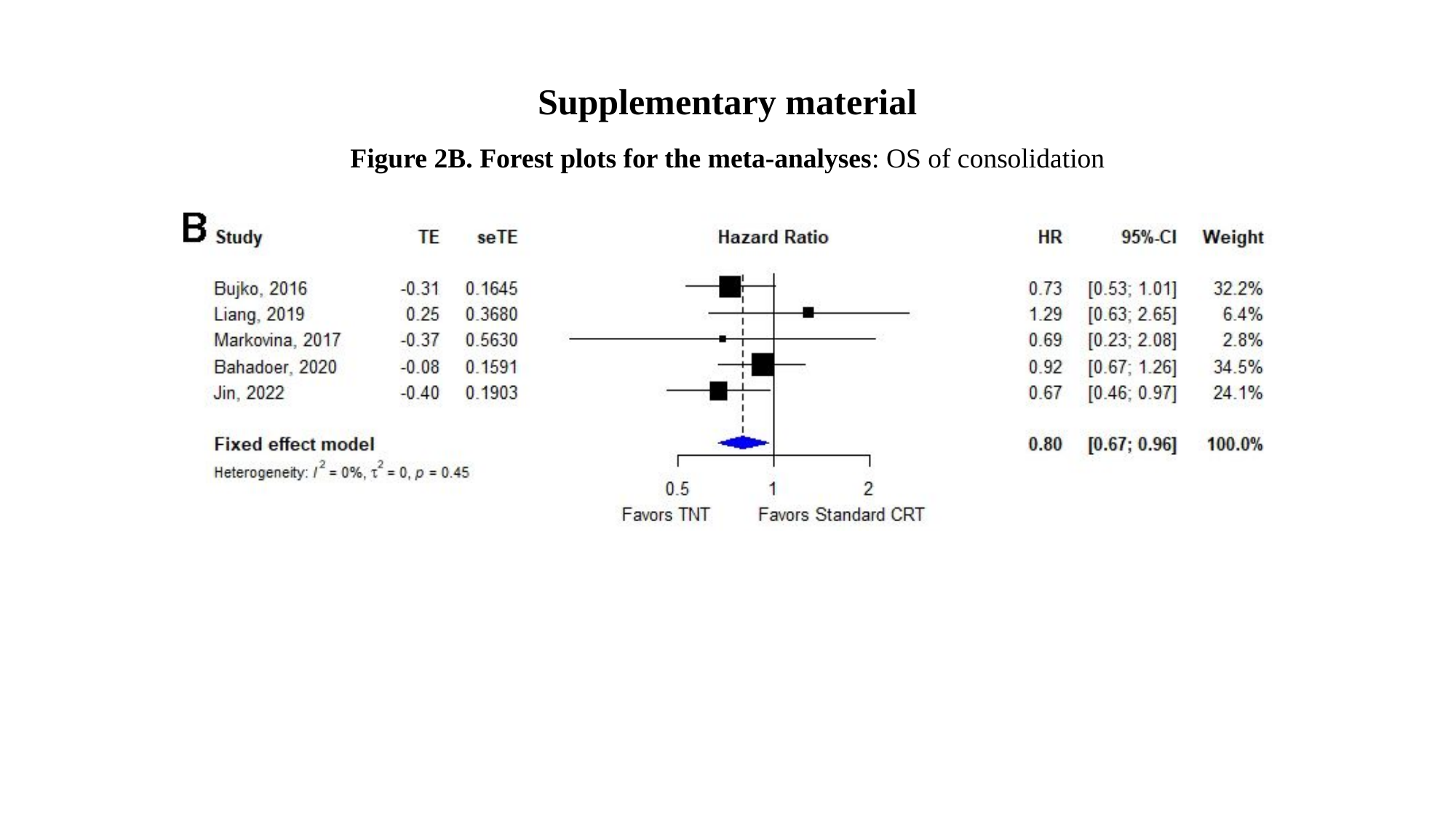

Supplementary material
Figure 2B. Forest plots for the meta-analyses: OS of consolidation
